# Does filter pore size introduce bias in DNA sequence-based plankton community studies?

**DOI:** 10.3389/fmicb.2022.969799

**Published:** 2022-09-26

**Authors:** Guolin Ma, Ramiro Logares, Yuanyuan Xue, Jun Yang

**Affiliations:** ^1^Aquatic EcoHealth Group, Fujian Key Laboratory of Watershed Ecology, Ningbo Observation and Research Station, Key Laboratory of Urban Environment and Health, Institute of Urban Environment, Chinese Academy of Sciences, Xiamen, China; ^2^College of Life Science, Fujian Agriculture and Forestry University, Fuzhou, China; ^3^Institute of Marine Sciences (ICM), Spanish National Research Council (CSIC), Barcelona, Spain; ^4^Zhejiang Key Laboratory of Urban Environmental Processes and Pollution Control, CAS Haixi Industrial Technology Innovation Center in Beilun, Ningbo, China

**Keywords:** eukaryotic plankton, environmental DNA, size-fractionated filtering, gene abundance, community composition, subtropical reservoir

## Abstract

The cell size of microbial eukaryotic plankton normally ranges from 0.2 to 200 μm. During the past decade, high-throughput sequencing of DNA has been revolutionizing their study on an unprecedented scale. Nonetheless, it is currently unclear whether we can accurately, effectively, and quantitatively depict the microbial eukaryotic plankton community using size-fractionated filtration combined with environmental DNA (eDNA) molecular methods. Here we assessed the microbial eukaryotic plankton communities with two filtering strategies from two subtropical reservoirs, that is one-step filtration (0.2–200 μm) and size-fractionated filtration (0.2–3 and 3–200 μm). The difference of 18S rRNA gene copy abundance between the two filtering treatments was less than 50% of the 0.2–200 μm microbial eukaryotic community for 95% of the total samples. Although the microbial eukaryotic plankton communities within the 0.2–200 μm and the 0.2–3 and 3–200 μm size fractions had approximately identical 18S rRNA gene copies, there were significant differences in their community composition. Furthermore, our results demonstrate that the systemic bias introduced by size-fractionation filtration has more influence on unique OTUs than shared OTUs, and the significant differences in abundance between the two eukaryotic plankton communities largely occurred in low-abundance OTUs in specific seasons. This work provides new insights into the use of size-fractionation in molecular studies of microbial eukaryotes populating the plankton.

## Introduction

Microbial eukaryotes are key components of aquatic ecosystems, where they can improve the water quality by naturally controlling the nutrient flux and energy transfer in aquatic communities (Cotner and Biddanda, 2002). As useful indicator organisms for assessing the ecosystem status, microbial eukaryotic plankton have been studied in diverse aquatic ecosystems (David et al., 2021; Yang J. et al., 2021). The discovery of many microbial eukaryotes has been aided by advances in instrumentation (e.g., flow cytometer), improved culturing techniques, and the development of molecular assays (Caron and Hu, 2019; Djurhuus et al., 2020). The continuous development and improvement of high-throughput sequencing (HTS) platforms have stimulated interest in the study of complex microbial communities (Jo et al., 2020; Garner et al., 2021). Currently, the most popular approach to studying microbial eukaryotic plankton community composition and dynamics is targeting the small subunit ribosomal RNA (SSU rRNA) gene (e.g., 18S rRNA gene) and sequencing it using HTS methods. The produced sequence data can provide an estimate of the relative abundance of each taxon in each sample. Environmental DNA (eDNA) metabarcoding typically associates HTS sequences with their taxonomy (Liu et al., 2020; Yang J. et al., 2021), allowing the characterization and biomonitoring of complex microbial communities. The eDNA approach is more convenient and comprehensive than conventional microscopy methods for analyzing microbial plankton across spatial and temporal scales, and therefore, for obtaining more comprehensive profiles of freshwater plankton communities (Banerji et al., 2018; Yang and Zhang, 2020). So far, many studies have used eDNA in the investigation of freshwater and marine microbial communities (Simon et al., 2015; Woodhouse et al., 2016; Xue et al., 2018; Lin et al., 2021; Mo et al., 2021).

There are inherent limitations in comparing HTS-generated relative abundances of taxa when analyzing microbiomes due to data compositionality (Gloor et al., 2017; Knight et al., 2018; Morton et al., 2019). The reason is that the increase in one taxon abundance causes an equivalent decrease across the remaining taxa, and it is hard to fully capture how individual microbial taxa differ among samples (Barlow et al., 2020; Yang C. Y. et al., 2021). Thus, in HTS-based analyses, the relative abundance of a taxon is dependent on the abundances of all other taxa, which can lead to high false-positive rates in taxon analyses (Weiss et al., 2017) and negative correlation biases in correlation-based analyses (Tsilimigras and Fodor, 2016). Apart from HTS, the real-time quantitative polymerase chain reaction (qPCR) is one of the most widely used methods of gene quantitation, which is also effective for the detection and quantification of specific eukaryotes from complex natural communities (Zhu et al., 2005). Characterizations of protistan communities based on HTS and qPCR can be affected by the varying rDNA copy numbers (Gong and Marchetti, 2019; Lavrinienko et al., 2020; Sandin et al., 2022), which is also a key trait in protists. All in all, these molecular methods have allowed a better characterization of the diversity of microbial eukaryotic plankton communities and extended our ability to describe them to an unprecedented resolution (Piwosz et al., 2020).

Cell size has been defined as a “master trait,” as it is shared by microorganisms across a given taxonomic group (Litchman et al., 2013). Eukaryotic planktonic microbes with different cell sizes might have fundamentally different characteristics and ecological roles in ecosystems (Clarke and Deagle, 2020). Molecular studies of microbial eukaryotic diversity have often used size-fractionated samples to separate unicellular eukaryotes from multicellular microorganisms, as well as to separate microbes with different cell sizes (de Vargas et al., 2015; Liu et al., 2017). Recent studies have revealed the community composition and spatiotemporal dynamic of eukaryotic plankton populating different size fractions (Woodhouse et al., 2016; Giner et al., 2019; Lin et al., 2021).

Lakes and reservoirs are excellent ecosystems for investigating microbial eukaryotic plankton (Zhang et al., 2020). In deep lakes, thermal stratification often facilitates chemical differences along the water column, resulting in the adaptation of microorganisms to different water layers (Boehrer and Schultze, 2008; Yu et al., 2014). In this study, we analyzed 18S rRNA gene abundances in size-fractionated plankton communities by qPCR and HTS. We collected plankton communities from five water layers in two subtropical reservoirs across four seasons. Our research focuses on the following hypotheses: (1) the 18S rRNA gene copies of the 0.2–200 μm size-fractionated eukaryotic plankton community is roughly equal to the sum of 0.2–3 and 3–200 μm fractions; (2) the community composition of the 0.2–200 μm size-fractionated eukaryotic plankton is roughly identical to the sum of the 0.2–3 and 3–200 μm fractions. We analyzed the abundance and composition differences between the two filtering treatments (Treatment 1: 0.2–200 μm and Treatment 2: 0.2–3 and 3–200 μm). Our results can contribute to understanding potential biases introduced by size-fractionation in studies of microbial eukaryotes.

## Materials and methods

### Sampling sites and filtration strategies

**FIGURE 1:**
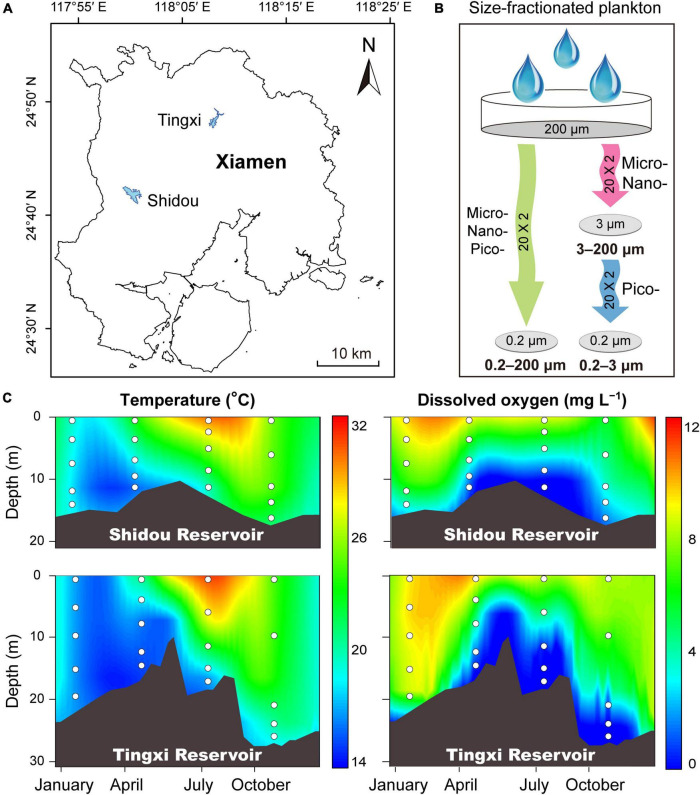
Sampling sites of the Shidou and Tingxi reservoirs in Xiamen city **(A)**, and size-fractionated filtering strategies **(B)**. Micro-, nano-, and pico-eukaryotic plankton: 0.2–200 μm; Micro- and nano-eukaryotic plankton: 3–200 μm; Pico-eukaryotic plankton: 0.2–3 μm. Depth-time profiles of water temperature and dissolved oxygen in the Shidou and Tingxi reservoirs, respectively **(C)**. White dots indicate the sampling depth.

Samples were collected from Shidou Reservoir (24°42′N, 118°00′E) and Tingxi Reservoir (24°48′N, 118°08′E), located in Xiamen, southeast China (Figure 1A). Both reservoirs are used for water supply, aquaculture, and irrigation. Details of these two reservoirs were described in our previous studies (Yang et al., 2016; Gao et al., 2022). Water samples were taken in January, April, July, and October during 2018. Stratification appeared based on water temperature and dissolved oxygen concentration in both the Shidou and Tingxi reservoirs during the observation term, while the mixing period occurred in January (Figure 1C). According to the water stratification conditions, five sampling depths were selected for plankton collection, represented by layer A (0.5 m depth), layer B (the average depth between layers A and C), layer C (thermal and hypoxic boundary depth), layer D (the average depth between layers C and E), and layer E (2 m above the bottom sediments), respectively. Water temperature and dissolved oxygen were measured *in situ* with a multi-parameter water quality analyzer (Hydrolab DS5, Hach Company, Loveland, CO, United States). After sampling, water samples were transported to the laboratory quickly. A 200 μm pore size nylon mesh was used to remove macroplankton and other large particles before water filtering (Liu et al., 2017). Next, a part of this water was filtered through 0.22 μm pore size polycarbonate membranes (47 mm diameter, Millipore, Bedford, MA, United States) to collect pico-, nano-, and micro-eukaryotic plankton cells within the 0.2–200 μm size fraction. The remaining water was filtered firstly through 3 μm pore size polycarbonate membranes (47 mm diameter, Millipore, Bedford, MA, United States) to collect nano- and micro-eukaryotic plankton cells within the 3–200 μm size fraction, and then filtered sequentially through 0.22 μm pore size polycarbonate membranes to obtain pico-eukaryotic plankton cells within the 0.2–3 μm size fraction (Figure 1B). The filtration volume ranged from 300 to 900 mL. Specifically, at the beginning of filtering, 300 ml of water sample was added to each filter membrane firstly and recorded the filtering time until completely filtered. If the time exceeded 30 min, we finished the collection of the filter membrane sample; otherwise, added another 50 ml of water sample successively until the filtering time reached 30 min, and the longest filtering time did not exceed 60 min. All these procedures were designed to ensure that each membrane reaches a critical load to collect enough plankton. A total of 120 filtered samples were acquired and the membranes were stored at –80°C until DNA extraction.

### DNA extraction and real-time quantitative PCR

The DNA of microbial eukaryotic plankton communities was extracted directly from the membranes using the FastDNA SPIN Kit (MP Biomedicals, Solon, OH, United States) according to the manufacturer’s instructions. The eukaryotic plankton was quantified by targeting the 18S rRNA gene. A pair of universal primers, 1380F (5′-CCCTGCCHTTTGTACACAC-3′) and 1510R (5′-CCTTCYGCAGGTTCACCTAC-3′) were used to amplify the eukaryotic 18S rDNA V9 region (Amaral-Zettler et al., 2009). Each 20 μL reaction mixture contained 10 μL of 2 × *PerfectStart*™ Green qPCR SuperMix, 0.5 μM of forward and reverse primers, 1 μL of DNA template and 8 μL RNase-free water. Negative controls contained the same mixtures with 1 μL of sterile water instead of the DNA template. Each plankton DNA sample was amplified in triplicates for 40 cycles of 30 s at 94°C, 15 s at 60°C, and 10 s at 72°C using an ABI Q6 Real-Time PCR System (Life Technologies, Applied Biosystems, Foster City, CA, United States). The DNA standards were prepared and run during each qPCR reaction to generate standard curves (*r*^2^ > 0.99). All qPCR runs yielded amplification efficiencies between 95% and 105%. The cycle number at which the fluorescence signal crosses a certain threshold (threshold cycle [Ct]) was noted. This Ct value is proportional to the logarithm of the target DNA concentration in the assay. From a dilution series of DNA amount corresponding to a known DNA concentration, a standard curve was produced. Combined with qPCR results, we calculated the 18S rRNA gene copies per liter of water and called this the 18S rRNA gene absolute abundance. The 18S rRNA gene absolute abundance of 0.2–3 and 3–200 μm size-fractionated plankton community is expressed as the sum of the 18S rRNA gene copies within 0.2–3 μm and 3–200 μm plankton communities.

### Metabarcoding, sequencing and bioinformatics

The primer pair 1380F and 1510R with barcodes were used to amplify the V9 region of the eukaryotic 18S rRNA gene (Hadziavdic et al., 2014; Xue et al., 2018). Each plankton DNA sample and negative controls were run in triplicates. The PCR reaction mixture contained 10 μL of Phusion High-Fidelity PCR Master Mix (New England Biolabs, Beverly, MA, United States), 0.5 μM of each primer, 1 μL of DNA template and 8 μL RNase-free water. The reactions included an initial denaturation at 95°C for 5 min, followed by 30 cycles of 30 s at 95°C, 30 s at 55°C, and 30 s at 72°C. At the end of the amplification, the amplicons were subjected to a final 5 min extension at 72°C. The triplicate PCR products for each of 120 samples were mixed in equimolar amounts and were confirmed after running in 2% agarose gel, and then isolated and purified using GeneJET Gel Extraction Kit (Thermo Fisher Scientific, Waltham, MA, United States). Sequencing libraries were generated using the NEB Next Ultra DNA Library Prep Kit for Illumina (New England Biolabs, Beverly, MA, United States) according to the manufacturer’s instructions. The library quality was evaluated using a Qubit 4.0 Fluorometer (Thermo Fisher Scientific, Waltham, MA, United States). Finally, the library was sequenced on the Illumina X Ten platform (Illumina Inc., San Diego, CA, United States) using a 150 bp paired-end protocol (Mo et al., 2021).

The paired-end V9 region of 18S rRNA gene sequences was processed using VSEARCH v.2.14.1 (Rognes et al., 2016). Chimeras were removed with default settings, and the unoise3 algorithm was used to identify operational taxonomic units (OTUs) at the 97% sequence similarity threshold (Nearing et al., 2018). Representative sequences were assigned taxonomy using the sintax algorithm with a cutoff value of 0.8 against the protist ribosomal reference database (PR2) in VSEARCH (Guillou et al., 2013). To minimize the inclusion of sequencing errors, the OTUs with less than 10 reads were excluded from the dataset before performing downstream analyses (Liu et al., 2017). The final total dataset retained 16,472 OTUs. We used a randomly selected subset of 209,120 sequences from each sample to normalize sequencing effort across samples based on MOTHUR v.1.39.5 (Schloss et al., 2009).

### Statistical analyses

The non-parametric Mann–Whitney *U* test was used to compare the difference in eukaryotic 18S rRNA gene copy abundance between the 0.2–200 μm and 0.2–3 and 3–200 μm eukaryotic plankton communities using SPSS v22.0 (IBM Corp., Armonk, NY, United States). The analysis of similarities (ANOSIM) was used to evaluate the differences between groups, with the global R representing the separation degree of between-group and within-group mean rank similarities. *R* = 0 indicates no separation, whereas *R* = 1 indicates complete separation (Clarke and Gorley, 2015). A non-metric multidimensional scaling (NMDS) ordination was used to investigate differences in microbial eukaryotic communities within different size fractions in R, using the “vegan” package (R Core Team, 2020). To characterize the beta-diversity of microbial eukaryotic plankton communities, we constructed Bray-Curtis dissimilarity matrices based on the Hellinger transformed read number. To compare the OTUs number among different communities, we constructed a Venn diagram using the “Venn-Diagram” package in R (R Core Team, 2020).

## Results

### Differences in 18S rRNA gene abundance between two filtering treatments

**FIGURE 2:**
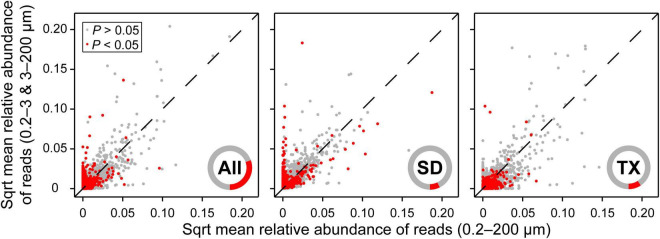
Comparison of relative abundance of OTUs between 0.2–200 μm and 0.2–3 and 3–200 μm eukaryotic plankton fractions. More than two-thirds of OTUs (69.58%) showed no significant difference in the relative abundance of reads between the two size-fractionated plankton communities in both reservoirs. The mean relative abundance of reads was square root transformation (Sqrt). Statistical analysis is the non-parametric Mann–Whitney *U* test. Data are presented as the mean of 40 replicates for 16472 OTUs in both reservoirs, 20 replicates for 14739 OTUs in the Shidou Reservoir and 20 replicates for 14540 OTUs in the Tingxi Reservoir, respectively. The operational taxonomic units (OTUs) were defined at 97% sequence similarity threshold. Only 5010 (30.42%), 1072 (7.27%), and 1358 (9.34%) OTUs were significant differences between the two size-fractionated plankton communities in both reservoirs (All), Shidou Reservoir (SD) and Tingxi Reservoir (TX), respectively. The inner red/gray cycles show the composition proportion with/without statistical differences, which more intuitively reflects the proportion with significant differences between the 0.2–200 μm and 0.2–3 and 3–200 μm size-fractionated eukaryotic plankton.

**FIGURE 3:**
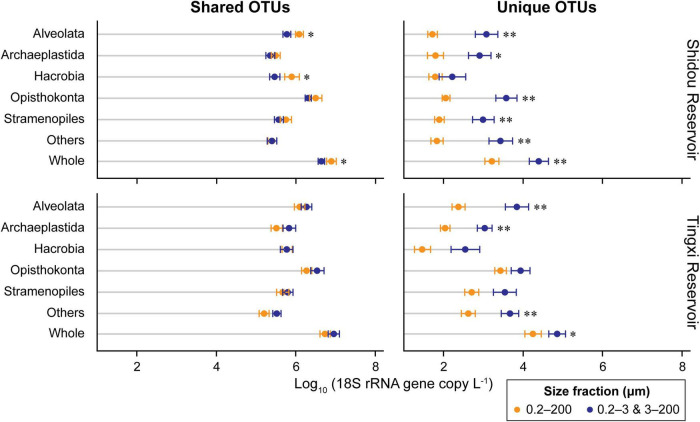
Comparison of 18S rRNA gene copy abundance of different supergroups between two size-fractionated plankton communities. The OTUs were defined at 97% sequence similarity threshold. Statistical analysis is non-parametric Mann–Whitney *U* test (*P < 0.05, ^**^P < 0.01). The error bar represents the standard error of OTU occurrences in all samples. Others includes Amoebozoa, Apusozoa, Excavata, and Rhizaria.

There were 14,739 and 14,540 plankton OTUs retrieved from the Shidou and Tingxi reservoirs, respectively (Supplementary Figure S1). The abundance of 18S rRNA gene for 69.58% (11462/16472), 92.73% (13667/14739) and 90.66% (13182/14540) OTUs was not significantly different between the two filtering treatments (0.2–200 μm *vs.* 0.2–3 and 3–200 μm) in both reservoirs, Shidou Reservoir and Tingxi Reservoir, respectively (Figure 2). Although the mean abundance in 30.42% OTUs (5010/16472) had a significant difference between the two size-fractionated eukaryotic plankton communities, all these OTUs only represented < 5% of the total reads (Figure 2), and most of them occurred as low-abundance OTUs (Table 1). More than half of the OTUs were shared (ca. 67.48% of the total OTUs in the Shidou Reservoir and 59.92% of those in the Tingxi Reservoir) (Supplementary Figure S1). The shared OTUs included the OTUs shared between the eukaryotic plankton communities with size fractions of 0.2–3 μm and 0.2–200 μm, 3–200 μm and 0.2–200 μm, also the OTUs shared among 0.2–3 μm, 3–200 μm and 0.2–200 μm size-fractionated eukaryotic communities. However, the systemic bias induced by different filtrations was more significant in the unique OTUs than in shared OTUs (Figure 3). Specifically, for the unique OTUs, the 18S rRNA gene abundance exhibited higher values in 0.2–3 and 3–200 μm size fractions than in the 0.2–200 μm size fraction (Figure 3). In the Shidou Reservoir, there were about 67.48% (9946/14739) shared OTUs for the two size-fractionated eukaryotic plankton communities; while 91.24% (9946/10901) for the 0.2–200 μm size fraction and 72.16% (9946/13784) for the 0.2–3 and 3–200 μm size fraction, respectively (Table 2 and Supplementary Figure S1). Tingxi Reservoir exhibited similar results for shared OTUs. More importantly, these shared OTUs contributed more than 95% of the reads in the two reservoirs for each size-fractionation treatment (Table 2).

**TABLE 1:** The OTUs that are different (*P* < 0.05) and non-significantly different (*P* ≥ 0.05) between the 0.2–200 μm and 0.2–3 and 3–200 μm eukaryotic plankton communities in both reservoirs, Shidou Reservoir and Tingxi reservoir, respectively.

OTUs relative abundance	Shidou and Tingxi reservoirs		Shidou reservoir		Tingxi reservoir	
	*P* < 0.05 (5,010 OTUs)	*P* ≥ 0.05 (11,462 OTUs)	*P* < 0.05 (1,072 OTUs)	*P* ≥ 0.05 (13,667 OTUs)	*P* < 0.05 (1,358 OTUs)	*P* ≥ 0.05 (13,182 OTUs)
≥1%	1 (0.01%)	14 (0.08%)	5 (0.03%)	6 (0.04%)	0 (0%)	16 (0.11%)
0.1% ∼ 1%	13 (0.08%)	108 (0.65%)	24 (0.16%)	110 (0.75%)	7 (0.05%)	100 (0.69%)
0.01% ∼ 0.1%	123 (0.75%)	497 (3.02%)	130 (0.88%)	424 (2.88%)	74 (0.51%)	508 (3.49%)
0.001% ∼ 0.01%	659 (4.00%)	1,387 (8.42%)	403 (2.74%)	1,408 (9.55%)	353 (2.43%)	1,463 (10.06%)
<0.001%	4,214 (25.58%)	9,456 (57.41%)	510 (3.46%)	11,719 (79.51%)	924 (6.35%)	11,095 (76.31%)

The operational taxonomic units (OTUs) were defined at 97% sequence similarity threshold. The percentage of OTU number is given in parentheses.

**TABLE 2:** The OTUs and sequences that are shared and unique between the 0.2–200 μm (*n* = 20) and 0.2–3 and 3–200 μm (*n* = 20) eukaryotic plankton communities from Shidou and Tingxi reservoirs.

	0.2–200 μm (shared)	0.2–200 μm (unique)	0.2–3 and 3–200 μm (shared)	0.2–3 and 3–200 μm (unique)
**Shidou**				
OTU	9,946 (91.24%)	955 (8.76%)	9,946 (72.16%)	3,838 (27.84%)
Sequence	262,448,030 (99.95%)	144,048 (0.05%)	122,689,638 (95.79%)	5,397,936 (4.21%)
**Tingxi**				
OTU	8,713 (82.03%)	1,909 (17.97%)	8,713 (68.98%)	3,918 (31.02%)
Sequence	216,087,304 (98.38%)	3,563,669 (1.62%)	376,846,508 (95.15%)	19,191,829 (4.85%)

The operational taxonomic units (OTUs) were defined at 97% sequence similarity threshold. The OTUs with ≤ 10 sequences were removed. The relative contributions (percentages) of shared or unique OTUs to total OTUs in each size fraction are given in parentheses.

**FIGURE 4:**
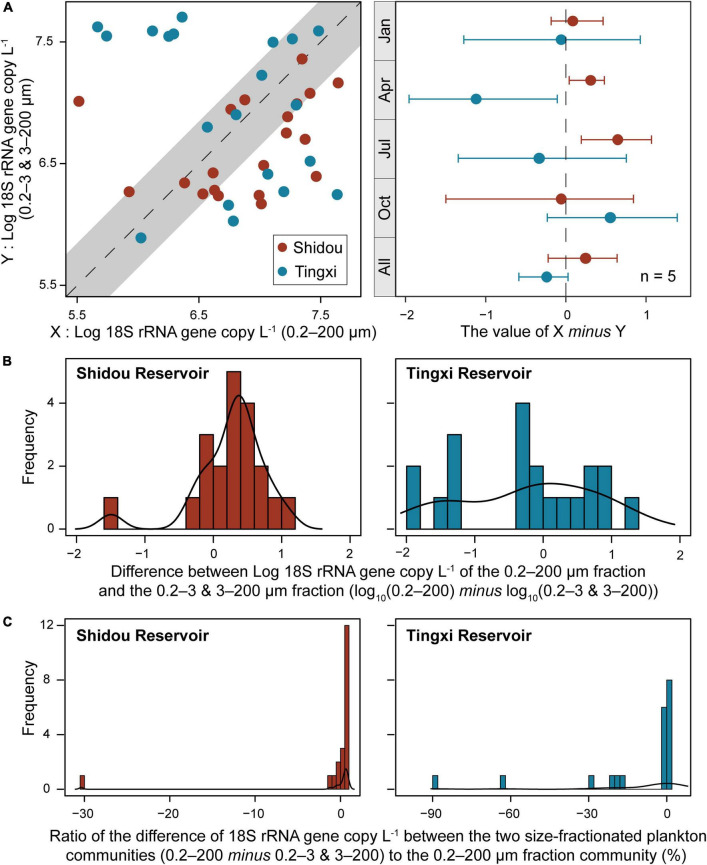
Comparison of the 18S rRNA gene abundance between the two size-fractionated plankton communities (with 95% confidence interval) and their seasonal difference (the error bar represents the standard error along with five water layers, *n* = 5) **(A)**. Histogram showing the distribution frequency of log10 18S rRNA gene copy of the 0.2–200 μm minus 0.2–3 and 3–200 μm size fractions **(B)**, and the ratio of the difference to 0.2–200 μm fraction **(C)**. The difference of 18S rRNA gene abundance between the two size-fractionated plankton communities was less than 50% of the 0.2–200 μm microbial eukaryotic community for 95% of the total samples.

**FIGURE 5:**
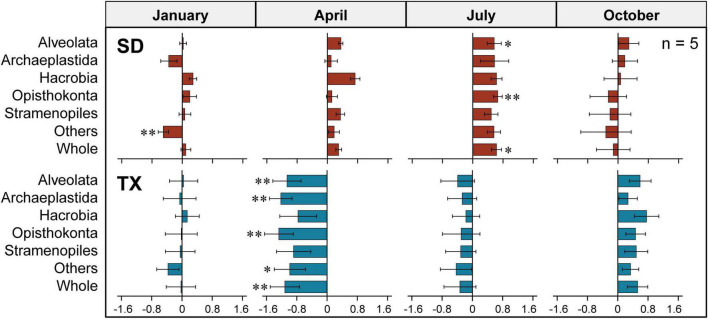
Differences of the 18S rRNA gene copy abundance between the two size-fractioned plankton communities at the supergroup level in Shidou (SD) and Tingxi (TX) reservoirs across four seasons, respectively. Size fractions: 0.2–200 μm *vs.* 0.2–3 and 3–200 μm. The data were log10-transformed. The error bar represents the standard error (*n* = 5). Statistical analysis is non-parametric Mann–Whitney *U* test (*P < 0.05, ^**^P < 0.01). Others includes Amoebozoa, Apusozoa, Excavata, and Rhizaria.

The absolute abundance of 18S rRNA gene copies in the three different size-fractionated planktonic communities (0.2–200 μm, 0.2–3 μm, and 3–200 μm) varied from 1.6 × 10^5^ to 4.4 × 10^7^ copies L^–1^ in the Shidou Reservoir, and 2.0 × 10^5^ to 4.9 × 10^7^ copies L^–1^ in the Tingxi Reservoir, respectively (Supplementary Figure S2). The two filtering treatments (0.2–200 μm *vs.* 0.2–3 and 3–200 μm) had roughly the same number of 18S rRNA gene copies in the Shidou Reservoir in January, April and October; and in the Tingxi Reservoir in January and July (Supplementary Figure S2 and Figure 4A). In the Shidou Reservoir, 40% of samples exhibited a higher 18S rRNA gene abundance in the 0.2–200 μm size fraction plankton community, and 55% of that were no significant difference between the two filtering treatments (0.2–200 μm *vs.* 0.2–3 and 3–200 μm). In the Tingxi Reservoir, 40% of samples had roughly the same 18S rRNA gene copy abundance, and 30% of samples exhibited a higher abundance in the 0.2–200 μm compared to 0.2–3 and 3–200 μm size-fractionated community (Figure 4A). Moreover, between the two size-fractionated plankton communities, the difference of 18S rRNA gene copies for 95% of the total samples was less than 50% of the 0.2–200 μm microbial eukaryotic community (Figure 4B), indicating the 18S rRNA gene copies of the two filtering treatments is roughly same across space and time. In addition, the significant difference of 18S rRNA gene abundance among different eukaryotic supergroups between the two filtering treatments only occurred in specific groups in specific seasons (i.e., July in the Shidou Reservoir, and April in the Tingxi Reservoir) (Figure 5).

### Differences in plankton community composition between two filtering treatments

The three size-fractionated eukaryotic plankton contributions to community were different at each supergroup level (Supplementary Figure S3). Both the Shidou and Tingxi reservoirs are characterized by plenty of OTUs belonging to 0.2–200 μm fraction plankton in almost all the supergroups (except for Apusozoa that with a lower OTUs richness and 18S rRNA gene abundance). Compared with the Tingxi Reservoir, only a small number of OTUs were found enriched in the Shidou Reservoir plankton within the 3–200 μm fraction but a large proportion of the OTUs harbored within the 0.2–3 μm fraction (Supplementary Figure S3). This result indicates that the Shidou Reservoir harbored a higher percentage of smaller eukaryotic plankton, while the larger individuals were enriched in the Tingxi Reservoir.

In both Shidou and Tingxi reservoirs, the two filtering treatments (0.2–200 μm *vs.* 0.2–3 and 3–200 μm) exhibited fewer variations in richness (OTUs) than in abundance (18S rRNA gene copies) (Supplementary Table S1), and the absolute abundance of the 18S rRNA gene fluctuated greatly among the five water layers and across four seasons (Supplementary Figures S2, S4). Additionally, Opisthokonta was the most abundant taxon compared with the other supergroups; it contributed 49.36% and 32.72% of the sequences in the 0.2–200 μm plankton community, while it contributed 43.67 and 46.20% of the sequences in the 0.2–3 and 3–200 μm size fractions in Shidou and Tingxi reservoirs, respectively (Supplementary Table S1 and Supplementary Figure S5). Alveolata was the second most abundant supergroup and represented a mean of 13.99% of the relative abundances in the Shidou Reservoir (0.2–200 μm: 13.51%; 0.2–3 and 3–200 μm: 14.46%) and 21.80% of that in the Tingxi Reservoir (0.2–200 μm: 23.53%; 0.2–3 and 3–200 μm: 20.06%) (Supplementary Table S1 and Supplementary Figure S5).

**FIGURE 6:**
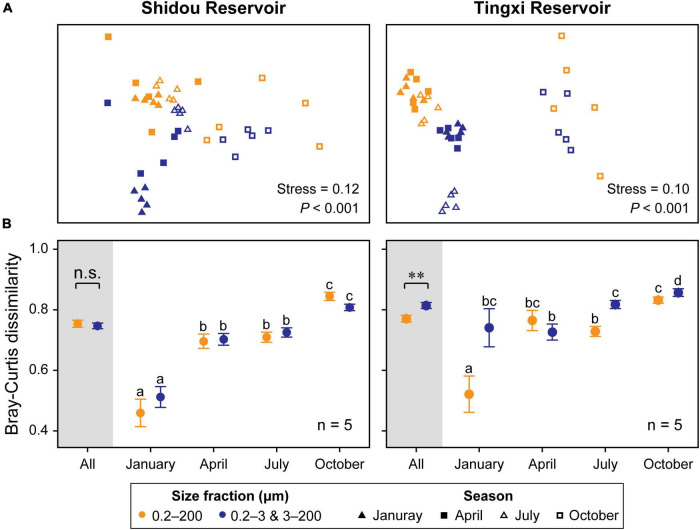
Non-metric multidimensional scaling (NMDS) ordination of eukaryotic plankton communities between two size fractions **(Color)** and across four seasons **(Shape)** in both reservoirs (Global R of size fractions is 0.150 and 0.214, while that of seasons is 0.514 and 0.545, respectively, in the Shidou and Tingxi reservoirs) **(A)**. Pairwise Bray–Curtis dissimilarity of different size-fractionated eukaryotic plankton communities. Data are mean ± standard error (SE), and the error bar represents the standard error along with five water layers (n = 5). Significant differences (*P* < 0.05) within-group are indicated with different letters by the non-parametric Mann–Whitney *U* test **(B)**. The small letters are used to distinguish whether there are statistical differences between the two filtering treatments (0.2–200 μm *vs.* 0.2–3 and 3–200 μm) in different seasons (January, April, July, and October). There is no significant difference between groups marked with the same letter, but there is a significant difference between groups marked with different letters.

The non-metric multidimensional scaling (NMDS) analysis showed that the eukaryotic plankton community composition was different between the 0.2–200 μm and the 0.2–3 and 3–200 μm fractions (Figure 6A), especially in January and July in the Shidou Reservoir and April in the Tingxi Reservoir (Supplementary Table S2).

### Spatiotemporal dynamics of two size-fractionated eukaryotic plankton communities

Seasonality was evident in both reservoirs for the two filtering treatments (0.2–200 μm *vs.* 0.2–3 and 3–200 μm) (Figure 6A), and the results of ANOSIM further corroborated that the eukaryotic plankton communities could be significantly distinguished across most seasons (Shidou Reservoir: from 0.304 to 0.848; Tingxi Reservoir: from 0.199 to 0.979), with an exception for the comparison between January and April (Supplementary Table S3). The two size-fractionated eukaryotic plankton communities did not show any significant difference in β-diversity in Shidou Reservoir, however, there were significant differences in Tingxi Reservoir, especially in January, July, and October (Figure 6B). Both reservoirs had the smallest Bray–Curtis dissimilarity between all pairs of samples in January, indicating a more similar community composition in winter caused by water mixing (Figures 1C, 6B).

## Discussion

Many studies have used size-fractionation to collect plankton of different size classes and showed community compositional variations among them. Such size-fractionated strategies have expanded our capacity to describe and compare different plankton communities with various resolutions (de Vargas et al., 2015; Liu et al., 2017; Lin et al., 2021). However, each methodological stage, from sampling to data analysis, can introduce biases; such biases can skew data sets by introducing changes in the observed relative abundance, and result in distorted observations of the true microbial composition within a sample (Costea et al., 2017; Yang C. Y. et al., 2021). Microbiome studies are particularly affected by these biases, especially in DNA sequence-based studies (Nearing et al., 2021). To determine the effects of different size-fractioning strategies on plankton community estimates, we assessed eukaryotic plankton abundance and community composition using DNA metabarcoding. Our results showed that microbial eukaryotic plankton communities between 0.2–200 μm and 0.2–3 and 3–200 μm size fractions had approximately identical 18S rRNA gene abundance in most cases, but there were significant differences in their composition. Besides, we found that the bias introduced by the two size-fractionating filtering strategies had more influence on unique OTUs than on shared OTUs, and the significant differences largely occurred in low-abundance taxonomic groups in specific seasons.

### Differences in 18S rRNA gene abundance between two filtering treatments

Our results showed that the 18S rRNA gene absolute abundance of the 0.2–200 μm eukaryotic plankton was roughly equal to the sum of 0.2–3 μm and 3–200 μm size fractions in most cases (Figures 2, 4B,C). Remarkably, there was a coherence of 18S rRNA gene abundance between the 0.2–200 μm and the 0.2–3 and 3–200 μm size fractions in the Shidou Reservoir, in contrast to the Tingxi Reservoir. This might be a characteristic difference between the two reservoirs. Specifically, Shidou Reservoir had high algal biomass due to the long-term dominance of a bloom-forming and toxic cyanobacterium (*Raphidiopsis raciborskii*) from September 2017 to November 2018 (Gao et al., 2022). The positive correlation between cyanobacteria abundance and other bacterioplankton in the water column has been shown by Woodhouse et al. (2016). Although more than half of the OTUs were shared by the two eukaryotic plankton communities, which may be caused by the eukaryotic plankton cells lysis and release of DNA into the water (Dell’Anno and Danovaro, 2005; Nagler et al., 2018). There still were a few significant differences that occurred in specific seasons.

Collectively, there are two main reasons for the differences in the 18S rRNA gene between the two filtering treatments. First, filtration might cause the loss of some microorganisms. Specifically, in the size-fractionation filtering, the cell morphology of microorganisms changed due to the negative pressure (Cavalier-Smith, 1997; Cavalier-Smith and Chao, 2004; Logares et al., 2014). After that, they would pass through the 3 μm even 0.22 μm pore size membrane, resulting in the loss of cell abundance. Pore size relates to the filter’s ability to filter out microbes with certain sizes. For example, a 0.22 μm membrane will filter out particles with a diameter of 0.22 microns or larger from the filtration stream. These biases might further lead to the underestimation of gene abundances in eukaryotic plankton communities. Second, microorganisms might accumulate on the membrane. As water filtering proceeded, a “filter cake layer” appeared on the pore size membranes, which is caused by larger microorganisms (Zheng et al., 2015). Accordingly, small cell plankton may be stuck on the filter membranes (Hasegawa et al., 2003). There were microbial accumulation on the filter membranes both in one-step and two-step filtering. However, microbes were retained more in the two-step filtration than in the one-step due to the longer (double) duration of water filtering. Furthermore, as the sequencing depth increased in the two-step filtration (0.2–3 and 3–200 μm) (Supplementary Figure S6), more rare OTUs with lower abundance will be obtained (Liu et al., 2017), which can lead to gene abundance differences between the two size-fractionated plankton communities in unique OTUs.

### Differences in plankton community composition between two filtering treatments

Our second hypothesis was that the community composition of the two size-fractionated eukaryotic plankton was identical. However, the community composition was significantly different between the 0.2–200 μm and the 0.2–3 and 3–200 μm size fractions, although more than half of the OTUs were shared by both fractions in Shidou and Tingxi reservoirs, respectively (Table 2 and Supplementary Figure S1). This is probably because the negative pressure filtering would cause eukaryotic plankton cells to break, and their cell-free DNA would attach to the filter membranes. According to Morrison et al. (2017), water stratification influenced the dominant taxa of the plankton community. Similarly, the differential dominance of eukaryotic microorganisms in different water layers (Supplementary Figure S4), further leads to the differences in community composition between the two filtering treatments (0.2–200 μm and 0.2–3 and 3–200 μm). In addition, we infer that the traits of abundant taxa also differentiate the community compositions between two size-fractionated plankton, due to their higher OTU richness and 18S rRNA gene abundance. For example, the flattened inner membrane complex of Apicomplexa (Alveolata), which evolved in association with fusion, is an adaptation for penetrating the host and gliding motility (Cavalier-Smith and Chao, 2004). Also, Opisthokonta is characterized by a single posterior cilium in their unicellular motile stage and by (non-discoid) flattened plate-like mitochondrial cristae (Cavalier-Smith, 1997). Their deformable morphological structure allows them to change their morphology and freely pass through the filter membranes.

As previous studies have shown, the apparent seasonal variations in microbial plankton communities have been shown in diverse ecosystems, including marine (Djurhuus et al., 2020) and freshwater environments (Simon et al., 2015; Salmaso et al., 2020). Most aquatic organisms have seasonal variation in community composition (de Vargas et al., 2015; Simon et al., 2015; Lin et al., 2021). In our study, the temporal dynamic of the microbial eukaryotes showed a strong seasonality at different taxonomic levels (Supplementary Figure S4 and Supplementary Table S3). Besides, the unique OTUs showed more significant differences between the two filtering treatments (0.2–200 μm *vs.* 0.2–3 and 3–200 μm), yet their relative abundance was much lower than that of shared OTUs. Lynch and Neufeld (2015) have pointed out that rare subcommunities are vital to shaping microbial community structure. In addition, the “rare biosphere” may contribute to the majority of the total richness in microbial communities (Sogin et al., 2006; Nyirabuhoro et al., 2020). Therefore, compared with previous studies (Staley et al., 2013; Logares et al., 2014), our results have illustrated the differences in the composition of different size-fractionated eukaryotic plankton communities largely due to the unique OTUs, although their abundances were very low.

There are still some inherent limitations in our study. For example, the use of a short region of 18S rRNA gene instead of the whole gene tends to exclude some taxa (Maritz et al., 2019). Besides, the choice of universal primers targeting the 18S rRNA gene might result in inaccurate estimates of the eukaryotic plankton community (Engelbrektson et al., 2010; Harder et al., 2016). The rRNA gene copy number varies from one to thousands in eukaryotic genomes and has a greater interspecific and intraspecific variation than in prokaryotic genome (de Vargas et al., 2015; Harder et al., 2016; Lavrinienko et al., 2020), which may also lead to the overestimation of microbes that are present in a sample as well as their proportions (Louca et al., 2018). Moreover, the rRNA gene copy number variability of the prevalent species hinders the accurate translation of metabarcoding data into relative abundance and of qPCR data into their corresponding cell numbers (Lajeunesse and Thornhill, 2011). Fortunately, the rRNA gene copy number per genome is significantly and positively correlated with the plankton size or biovolume (de Vargas et al., 2015; Lavrinienko et al., 2020), therefore we can roughly estimate the plankton biomass based on the rRNA gene copy number. More noticeably, the effects of processing methods in protocols often exceed the biological effects underlining the importance of minimizing these biases (Sinha et al., 2017). Nevertheless, these potential sources of error have not been systematically examined in the development of approaches in microbial eukaryotic plankton studies.

In this study, we have compared the two eukaryotic plankton communities with sizes that overlapping and mutually contained (e.g., 0.2–200 μm *vs*. 0.2–3 and 3–200 μm), trying to illustrate the impact of size-fractionated filtering strategies on the plankton results. Here, we provide evidence that the commonly used size-fractionation particularly affected the low-abundance taxa of the eukaryotic plankton community in specific seasonal eDNA samples.

## Conclusion

We compared the 18S rRNA gene absolute abundance and the community composition of the two filtering treatments (0.2–200 μm *vs.* 0.2–3 and 3–200 μm) from two subtropical reservoirs using qPCR and HTS. Our results reflect the quantitative changes of eukaryotic plankton within two filtering treatments. Both size-fractionated plankton communities had approximately equal 18S rRNA gene abundance in most cases but exhibited dissimilar community composition across four seasons. This suggests that the systemic bias in unique OTUs introduced by different filtration strategies has influenced the consistency between the 0.2–200 μm and 0.2–3 and 3–200 μm size fractions, but the general seasonal patterns were highly similar. In addition, we demonstrated that the difference in temporal scale (seasons) considerably exceeds that in spatial scale (water layers and size fractions). Therefore, we conclude that DNA-based size-fractionated filtering (i.e., 0.2–200 μm and 0.2–3 and 3–200 μm) cannot change the abundance of dominant taxa in most cases, but can alter the results of low-abundance taxa. This work provides new insights into the application of various size-fractionation filtering strategies in the DNA sequencing-based study of microbial eukaryotes.

## Data availability statement

The data presented in this study are deposited in the NCBI database, accession number: PRJNA770434.

## Author contributions

JY led and designed the research. YX performed the sample collection and the PCR. GM performed the qPCR, high-throughput sequencing, and bioinformatics. GM and JY analyzed the data and wrote the first draft of the manuscript. YX and RL reviewed and edited the manuscript. All authors contributed to and approved the final manuscript.
