# Supplementary material for: Does filter pore size introduce bias in DNA sequence-based plankton community studies?
Source: Front Microbiol. 2022 Sep 26;13:969799. doi: 10.3389/fmicb.2022.969799 (PMC9549009; doi:10.3389/fmicb.2022.969799)
Supplement: Supplementary file 1 [file Data_Sheet_1.docx]

**Journal: Frontiers in Microbiology**

*Supplementary materials of the article*

**Does filter pore size introduce bias in DNA sequence-based plankton community studies?**

Guolin Ma^1,2^, Ramiro Logares^3^, Yuanyuan Xue^1,4*^, Jun Yang^1,4*^

^1^*Aquatic EcoHealth Group, Fujian Key Laboratory of Watershed Ecology, Ningbo Observation and Research Station, Key Laboratory of Urban Environment and Health, Institute of Urban Environment, Chinese Academy of Sciences, Xiamen, China*

^2^*College of Life Science, Fujian Agriculture and Forestry University, Fuzhou, China*

^3^*Institute of Marine Sciences (ICM), Spanish National Research Council (CSIC), Barcelona, Spain*

^4^*Zhejiang Key Laboratory of Urban Environmental Processes and Pollution Control, CAS Haixi Industrial Technology Innovation Center in Beilun, Ningbo, China*

***Correspondence:**

Yuanyuan Xue, E-mail: [yyxue@iue.ac.cn](mailto:yyxue@iue.ac.cn); Jun Yang, E-mail: [jyang@iue.ac.cn](mailto:jyang@iue.ac.cn)

**The supplementary materials contain:**

⚫ 10 Pages

⚫ 6 Figures

⚫ 3 Tables


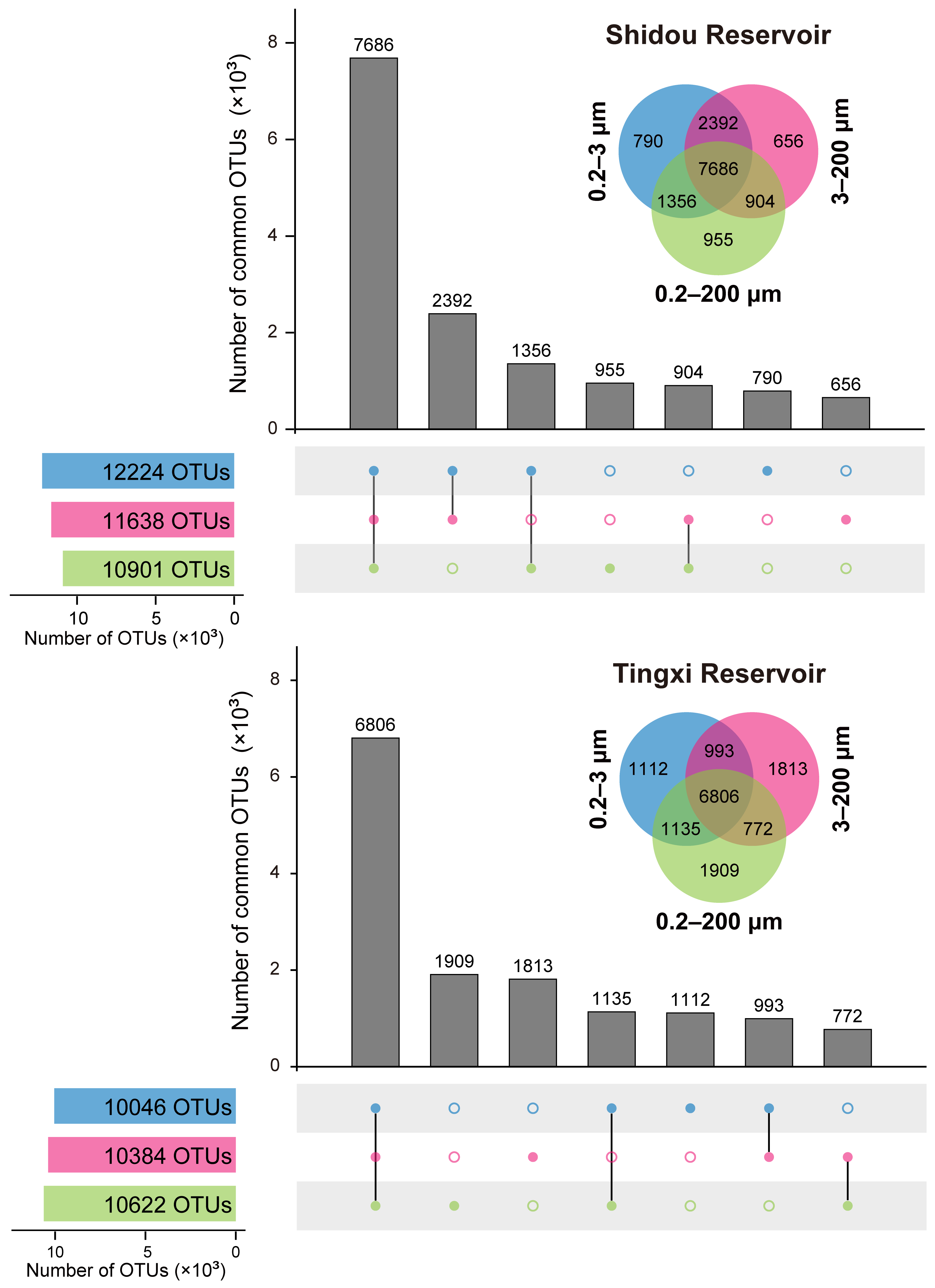


**Supplementary Figure S1** Distributions of the shared and unique eukaryotic OTUs among three size-fractionated plankton communities in the Shidou and Tingxi reservoirs, respectively. The operational taxonomic units (OTUs) were defined at 97% sequence similarity threshold. Green, blue, and pink colors indicate the 0.2–200 μm, 0.2–3 μm and 3–200 μm size-fractionated eukaryotic plankton communities, respectively.


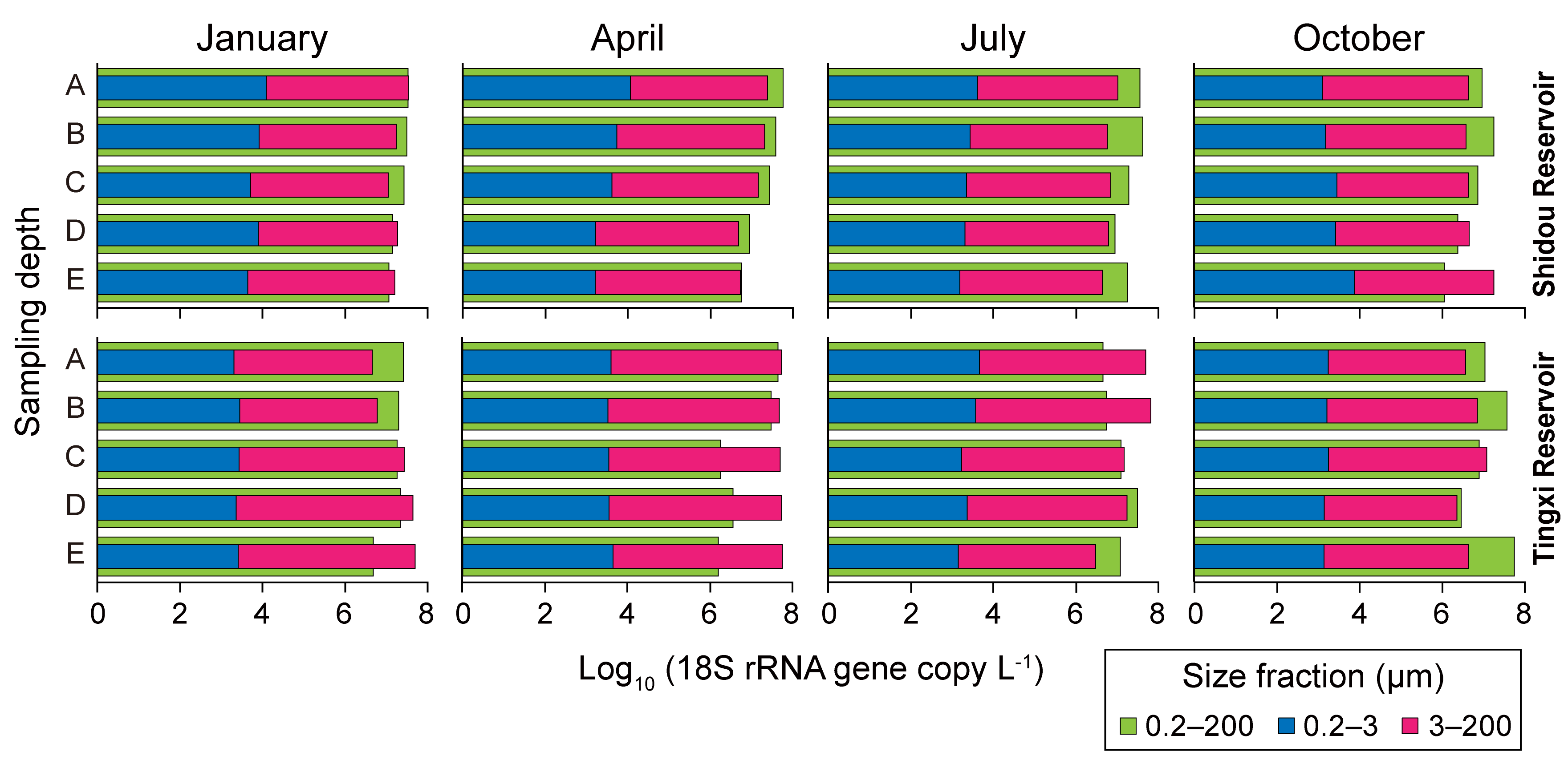


**Supplementary Figure S2** The 18S rRNA gene abundance of three different size-fractionated plankton communities from five water layers in the Shidou and Tingxi reservoirs, respectively. The data were log10-transformed. A, B, C, D, and E represent the five different layers from surface to bottom waters.

**
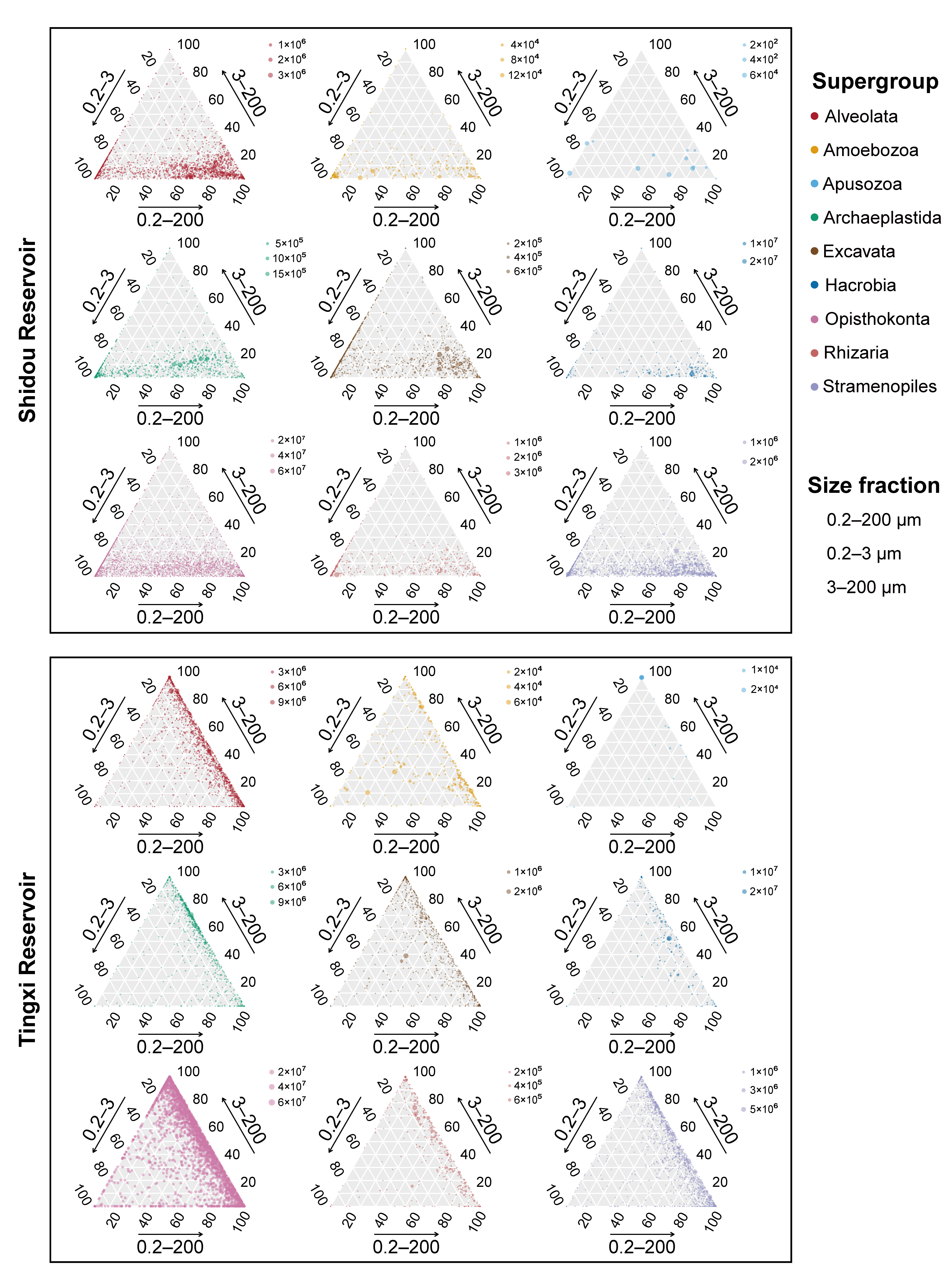
**

**Supplementary Figure S3** Ternary plots showing the 18S rRNA gene abundance in three size-fractionated plankton communities (0.2–200 μm, 0.2–3 μm and 3–200 μm) at the level of supergroup. Each dot represents an OTU. The size of each dot is proportional to the absolute abundance (copy L^-1^) and dots are colored according to different supergroups.


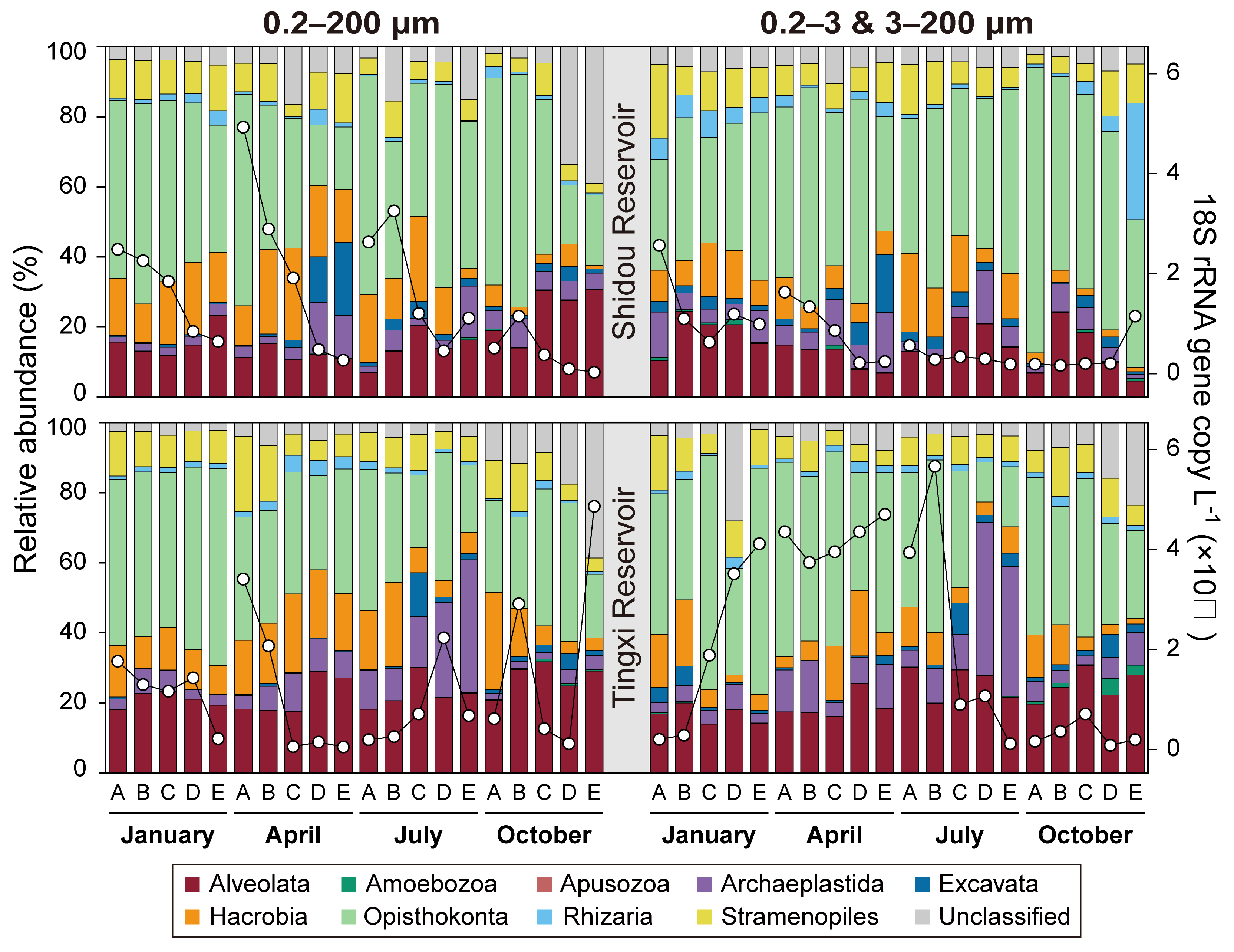


**Supplementary Figure S4** The community composition and 18S rRNA gene abundance of eukaryotic plankton with different size fractions from five water layers in the Shidou and Tingxi reservoirs, respectively. White dots exhibit the 18S rRNA gene copies of each sample. A, B, C, D, and E represent the five different layers from surface to bottom waters. Unclassified represents the eukaryotic taxa could not be discriminated at similarity ≥ 80%.


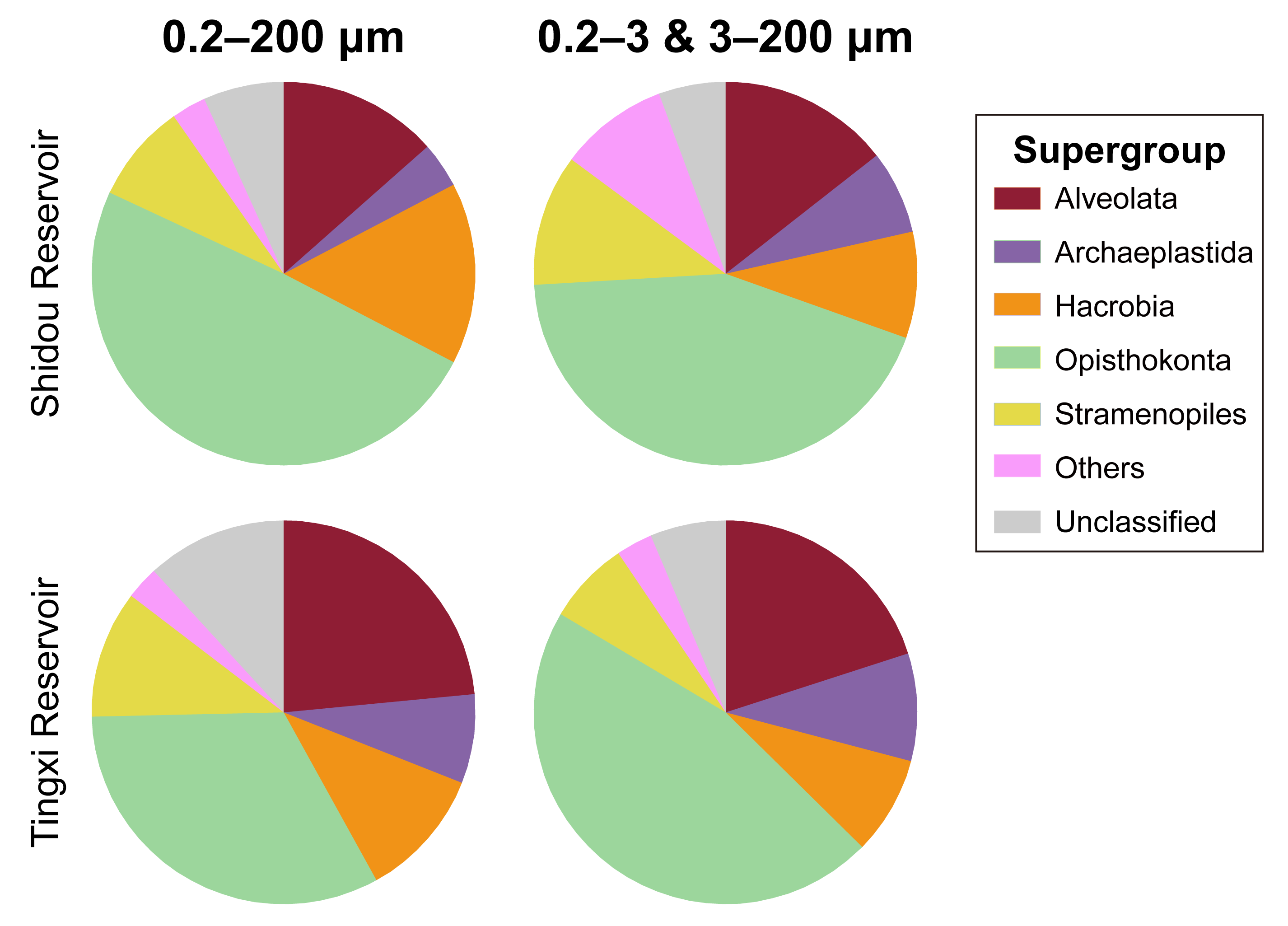


**Supplementary Figure S5** Community composition of the two size-fractionated eukaryotic plankton at the level of supergroup in the Shidou and Tingxi reservoirs, respectively. Others includes Amoebozoa, Apusozoa, Excavata and Rhizaria. Unclassified represents the eukaryotic taxa could not be discriminated at similarity ≥ 80%.

**
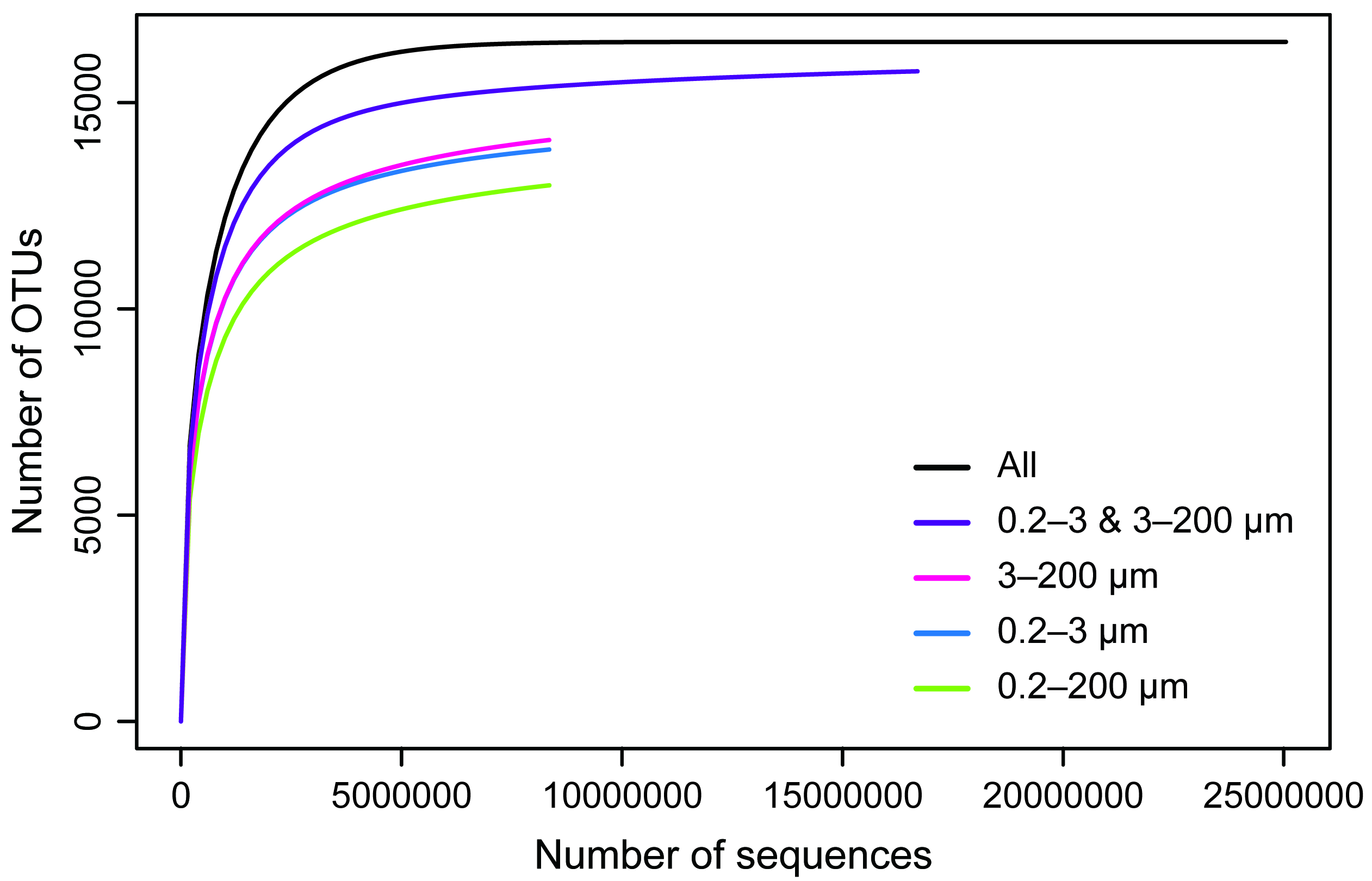
**

**Supplementary Figure S6** Rarefaction curves of similarity-based operational taxonomic units (OTU) at 97% sequence similarity level. Lines with different colors represent the eukaryotic plankton communities with different size fractions.

**Supplementary Table S1** Absolute abundances of taxonomic groups based on 18S rRNA gene copies between two filtering treatment plankton communities (0.2–200 μm and 0.2–3 & 3–200 μm) in the Shidou and Tingxi reservoirs, respectively.

| Supergroup | **Shidou Reservoir** | | | | |  | **Tingxi Reservoir** | | | | |
| --- | --- | --- | --- | --- | --- | --- | --- | --- | --- | --- | --- |
|  | 0.2–200 μm | |  | 0.2–3 & 3–200 μm | |  | 0.2–200 μm | |  | 0.2–3 & 3–200 μm | |
|  | OTUs | 18S rRNA  gene copies |  | OTUs | 18S rRNA  gene copies |  | OTUs | 18S rRNA  gene copies |  | OTUs | 18S rRNA  gene copies |
| Aleveolata | 1,249  (11.46%) | 35,484,551  (13.51%) |  | 1,558  (11.30%) | 18,517,838  (14.46%) |  | 1,309  (12.32%) | 51,684,248  (23.53%) |  | 1,716  (13.59%) | 79,436,599  (20.06%) |
| Archaeplastida | 586  (5.38%) | 10,058,868  (3.83%) |  | 785  (5.71%) | 9,020,047  (7.04%) |  | 649  (6.11%) | 16,498,000  (7.51%) |  | 748  (5.92%) | 35,872,917  (9.06%) |
| Hacrobia | 145  (1.33%) | 40,200,471  (15.31%) |  | 174  (1.26%) | 11,512,068  (8.99%) |  | 142  (1.34%) | 24,132,802  (10.99%) |  | 200  (1.58%) | 32,816,838  (8.29%) |
| Opisthokonta | 1,923  (17.72%) | 129,616,836  (49.36%) |  | 2,676  (19.41%) | 55,931,113  (43.67%) |  | 2,148  (20.22%) | 71,874,294  (32.72%) |  | 2,605  (20.62%) | 182,958,583  (46.20%) |
| Stramenopiles | 1,095  (10.04%) | 21,973,602  (8.37%) |  | 1,376  (9.98%) | 14,197,703  (11.08%) |  | 1,169  (11.01%) | 23,616,097  (10.75%) |  | 1,452  (11.50%) | 27,711,844  (6.99%) |
| Others | 1,128  (10.35%) | 7,572,947  (2.88%) |  | 1,697  (12.31%) | 11,776,371  (9.19%) |  | 1,190  (11.20%) | 6,115,818  (2.78%) |  | 1,550  (12.27%) | 12,122,641  (3.06%) |
| Unclassified | 4,766  (43.72%) | 17,684,803  (6.74%) |  | 5,518  (40.03%) | 7,132,434  (5.57%) |  | 4,015  (37.80%) | 25,729,714  (11.72%) |  | 4,360  (34.52%) | 25,118,915  (6.34%) |

The operational taxonomic units (OTUs) were defined at the 97% sequence similarity threshold.

The relative contributions (percentage) of OTUs and their sequences in each size fraction are given in parentheses.

Others includes Amoebozoa, Apusozoa, Excavata and Rhizaria.

Unclassified represents eukaryotic taxons that could not be discriminated at similarity ≥ 80%.

**Supplementary Table S2** Analysis of similarity (ANOSIM) statistics testing the difference of two size-fractionated eukaryotic plankton communities in Shidou and Tingxi reservoirs, respectively.

| 0.2–200 μm *vs.* 0.2–3 & 3–200 μm | Shidou Reservoir | |  | Tingxi Reservoir | |
| --- | --- | --- | --- | --- | --- |
|  | Global R | *P* |  | Global R | *P* |
| January | **0.668** | **0.008** |  | 0.048 | 0.183 |
| April | 0.100 | 0.198 |  | **0.488** | **0.008** |
| July | **0.464** | **0.024** |  | 0.220 | 0.127 |
| October | 0.092 | 0.198 |  | 0.108 | 0.206 |

The ANOSIM statistic R is calculated by the difference of the between-group mean rank similarities, thus it displays the degree of separation between groups. A higher R-value indicates the higher difference between groups.

Bold font means the significance at *P* < 0.05 level.

**Supplementary Table S3** Analysis of similarity (ANOSIM) statistics testing the spatiotemporal difference in eukaryotic plankton community from the Shidou and Tingxi reservoirs, respectively.

| Grouping by | Shidou Reservoir | |  | Tingxi Reservoir | |
| --- | --- | --- | --- | --- | --- |
|  | Global R | *P* |  | Global R | *P* |
| **Layer (5 layers)** | -0.074 | 0.990 |  | -0.084 | 0.998 |
| A *vs.* B | -0.099 | 0.944 |  | -0.119 | 0.990 |
| A *vs.* C | -0.068 | 0.791 |  | -0.073 | 0.849 |
| A *vs*. D | -0.033 | 0.588 |  | -0.073 | 0.849 |
| A *vs.* E | -0.022 | 0.549 |  | -0.059 | 0.779 |
| B *vs.* C | -0.088 | 0.866 |  | -0.086 | 0.895 |
| B *vs.* D | -0.065 | 0.788 |  | -0.074 | 0.848 |
| B *vs*. E | -0.072 | 0.835 |  | -0.059 | 0.778 |
| C *vs.* D | -0.085 | 0.896 |  | -0.116 | 0.975 |
| C *vs.* E | -0.097 | 0.949 |  | -0.091 | 0.914 |
| D *vs.* E | -0.124 | 0.999 |  | -0.103 | 0.968 |
| **Season (4 seasons)** | **0.514** | **0.001** |  | **0.545** | **0.001** |
| January *vs.* April | 0.149 | 0.065 |  | 0.091 | 0.129 |
| January *vs.* July | **0.519** | **0.001** |  | **0.330** | **0.006** |
| January *vs.* October | **0.848** | **0.001** |  | **0.960** | **0.001** |
| April *vs.* July | **0.304** | **0.001** |  | **0.199** | **0.029** |
| April *vs.* October | **0.698** | **0.001** |  | **0.942** | **0.001** |
| July *vs.* October | **0.790** | **0.001** |  | **0.979** | **0.001** |
| **Size fraction (0.2–200 μm *vs.* 0.2–3 & 3–200 μm)** | **0.150** | **0.002** |  | **0.214** | **0.001** |

The ANOSIM statistic R is calculated by the difference of the between-group mean rank similarities, thus it displays the degree of separation between groups. A higher R-value indicates the higher difference between groups.

A, B, C, D, and E represent five different layers from surface to bottom waters.

Bold font means the significance at *P* < 0.05 level.
